# Medicine in the Popular Press: The Influence of the Media on Perceptions of Disease

**DOI:** 10.1371/journal.pone.0003552

**Published:** 2008-10-29

**Authors:** Meredith E. Young, Geoffrey R. Norman, Karin R. Humphreys

**Affiliations:** 1 Department of Psychology, Neuroscience and Behaviour, McMaster University, Hamilton, Ontario, Canada; 2 Program for Educational Research and Development, (PERD), Department of Clinical Epidemiology and Biostatistics, McMaster University, Hamilton, Ontario, Canada; Copenhagen University Hospital, Denmark

## Abstract

In an age of increasing globalization and discussion of the possibility of global pandemics, increasing rates of reporting of these events may influence public perception of risk. The present studies investigate the impact of high levels of media reporting on the perceptions of disease. Undergraduate psychology and medical students were asked to rate the severity, future prevalence and disease status of both frequently reported diseases (e.g. avian flu) and infrequently reported diseases (e.g. yellow fever). Participants considered diseases that occur frequently in the media to be more serious, and have higher disease status than those that infrequently occur in the media, even when the low media frequency conditions were considered objectively ‘worse’ by a separate group of participants. Estimates of severity also positively correlated with popular print media frequency in both student populations. However, we also see that the concurrent presentation of objective information about the diseases can mitigate this effect. It is clear from these data that the media can bias our perceptions of disease.

## Introduction

*“The news media are not successful in telling us what to think, but they do succeed in telling us what to think about”* [1: p. 682].

Public health expenditure is on the rise, humans are living longer than ever before, medical therapies are increasingly saving lives, yet people view themselves as more vulnerable than ever before [2]. There is a discontinuity between the objective assessment of risk to an individual and an individual's subjective assessment of that same risk. When asked to rate the likelihood of death from a variety of causes, we tend to underestimate common causes and overestimate rare causes of death [3]: these estimates have little relationship to actual mortality statistics [4]–[5].

Individuals have at least two main sources of information regarding risk and, by extension, from which to base their judgments of risk: the media and interpersonal networks [6]. Interpersonal networks are, by their nature, idiosyncratic; thus, individual variation of available information should not lead to the systematic trends in estimation seen in these studies. By contrast, information provided through media sources may well lead to systematic over- or underestimates at a population level. In an investigation by Combs and Slovic, individuals' estimates of causes of mortality were not correlated with actual mortality statistics, but participants' estimates were strongly correlated with the frequency of print media reporting [4]. Consistent with this, Frost, Frank and Maibach found a poor association between the frequency of reporting in print media and actual risk and mortality rates [3], and Kristiansen found no relationship between the frequency of reporting deaths and the actual mortality rates [7].

This trend is not limited to mortality statistics. A relationship between media attention and public concern has been demonstrated in global warming [1], genetically modified foods [6], probability of disease [3], overall health [8] and health related accidents such as Creutzfeldt-Jakob disease [9], microbial illnesses [10], and drug effects [11]. In addition, media influences have been documented in political agenda setting [12], the risks associated with electromagnetic fields [13], genetic research [14], and stress reactions to domestic terrorism [15]–[20], international terrorism [21]–[27], and bioterrorist attacks [28]. The media tend to focus on rare and dramatic events. As a topic receives repeated coverage in the media, public attention is drawn towards that particular topic and away from competing sources of concern [2]. This dynamic relationship raises concerns regarding recent media-dominating topics such as national and international terrorism, newly emerging and re-emerging infectious diseases, and other rare but dramatic hazards [11].

This association between media frequency and public reaction is not benign, but can itself induce health consequences. Research conducted after the Oklahoma bombing showed that for children outside of directly affected areas, media exposure was a strong predictor of posttraumatic stress syndrome and stress reactions [15]–[20]. A similar pattern was seen after the September 11^th^ attack in New York, where several studies showed associations between viewing television coverage of the attack and self-reported posttraumatic stress symptomatology [20]–[27]. Similarly, the Chernobyl incident of 1986 induced considerably more stress-related disease than it did cancer [29], and suicide was the leading cause of death among Estonian clean-up workers [30].

Of course, the media have a role in disseminating information to warn the public about health concerns. In the case of Reyes' syndrome in children who had been treated with acetylsalicylic acid [6], the news media were pivotal in alerting the public. However, events that amplify or attenuate public concern are not easily predicted [6], and numerous examples exist where the amplification of the perceived risk was not ultimately accompanied by a commensurate risk increase such as; the Chernobyl Disaster [29], [31], cancer risk from cell phones [32], and anthrax outbreaks [28].

*“Unlike other infectious diseases, anthrax is not communicable, yet it virtually immobilized Washington, DC”* [28: p. 1084].

It is this particular manifestation of public perception that this paper explores – to what extent is public perception of infectious disease modulated by the high levels of popular media coverage in North America? The current literature focuses on ‘risk assessment’ and differential estimated rates of various events. Disproportionate media exposure may have effects on perceptions of disease other than estimates of prevalence, such as disease severity, and whether something is a disease at all. If increased media frequency can in fact alter perceptions of the concept of disease, or what is publicly recognized as a serious disease, then this has implications for many aspects of health decision making. The studies included in this paper investigate the effect of frequent news media exposure on perceived severity, disease–like status and prevalence of infectious diseases.

## Methods

### Experiment 1

#### Participants

Undergraduate students from the psychology participant pool at McMaster University (n = 52, 33 female; age range approximately 17–23) participated in this study for experimental course credit. The only criterion for participation was that English was spoken with at least near-native fluency. No information was collected about participants' media consumption habits. This study was approved by the McMaster University Research Ethics Board.

#### Study Design

Upon arrival at the laboratory, participants were orally briefed regarding the procedures of the experiment, and written consent was obtained. Participants were asked to complete a survey consisting of 10 different medical conditions, 5 of which were high media frequency conditions and 5 of which were low media frequency conditions. Participants were asked to make three different judgments on each of the 10 medical conditions. Participants were asked to judge the seriousness of the medical condition on a 10 point scale (where 1 was ‘not very serious’ and 10 was ‘very serious’), the likelihood that the condition described represented a disease on a 4 point scale (where 1 was ‘definitely not a disease’ and 4 was ‘definitely a disease’), and were asked to estimate the prevalence of the described condition (‘out of a sample of 1,000 of your cohort, estimate how many are likely to have the condition in the next year’). Estimated prevalence is often used as an indicator of perceived risk [4]–[5], however a total of three response scales were used in order to evaluate the possibility of a more complex change in the understanding of illnesses frequently reported in the media. Perhaps with the drastically increased reporting of such threats as SARS and avian flu, we will observe a differential treatment of high media diseases that is not limited to an increased reporting of prevalence, but indicates instead a more holistic shift in the conceptualization of these highly reported illnesses.

Participants were assigned randomly to either a low information or a high information condition. In the low information condition participants were required to make judgments based only on the name of the disorder. In the high information condition participants were required to make judgments based on the name of the disorder, followed by a short description that included information regarding the symptoms, prevalence, mode of transmission, and fatality of the condition. An example of the informational conditions can be seen in Table 1. This manipulation was included in order to evaluate the ability of immediately available information to mediate the perceptions of high media diseases.

**Table 1:** Informational Conditions: Example*.

Low information condition	West Nile Virus
High information condition	West Nile Virus: West Nile Virus is transmitted by a bite from an infected mosquito. 80% of people who do get infected will not show any symptoms. West Nile Virus symptoms can include headaches, nausea, vomiting, skin rash, high fever, headaches, neck stiffness, stupor, disorientation, tremors, muscle weakness, vision loss and paralysis. In 2005, there were 224 reported cases of West Nile Virus in Canada, 12 of the cases were fatal.

* Note: West Nile Virus is used as an example. A similar format was used for all diseases included in this paper.

The order of the presentation of medical conditions was counterbalanced across subjects. All diseases were entered into a Lexis Nexus database search for frequency within major North American print media sources for the 12 months preceding the completion of testing.

#### Analysis

A mixed design Analysis of Variance (ANOVA) was conducted, where the comparison of interest was between high and low media frequency diseases, and the within subject variable was the judgments made on each of the individual diseases. Amount of information provided was included as a between subjects independent variable.

### Experiment 2

The methods were identical to those of Study 1, with the exception of the participant pool and their compensation. Forty-three first-year medical students (25 female; approximate age range 21–29) voluntarily participated in this study, and the survey was conducted as an aspect of a course on research design. All participants consented to having their anonymized data analyzed for the purposes of research. This study was approved by the McMaster University Research Ethics Board.

#### Analysis

Data were analyzed using the same techniques as in Study 1. A mixed design Analysis of Variance (ANOVA) was conducted where the comparison of interest was between high and low media frequency diseases, the within-subject variable was the individual diseases, and information level was also included as a between-subjects variable.

### Experiment 3

Twelve graduate students (7 females; age range approximately 21–27) in the Department of Psychology, Neuroscience and Behaviour at McMaster University. Each pair of conditions was presented to participants, without disease labels, and they were asked to make a two-option forced choice decision regarding which of the two conditions was ‘worse’. Participants were encouraged to use their own metric for deciding which of the two disorders was more severe.

## Results

### Experiment 1

This study was designed to investigate the impact of the disease label and associated knowledge on judgments of severity, prevalence and disease-like status of both high media frequency and low media frequency diseases. For the purposes of this experiment, ten infection diseases drawn from the Centre for Disease Control database were used. Five were medical disorders that have been highly prevalent in the recent news media (anthrax, SARS, West Nile virus, Lyme disease and avian flu) and five were medical disorders that have not often been present in current news media (tularemia, human babesiosis, yellow fever, Lassa fever and hantavirus). High and low media frequency diseases were confirmed using a LexisNexus search of general news media. For the purposes of this study, popular news media included North American magazines and newspapers generally read or accessible to the public. Each of the ‘low media’ frequency diseases was chosen to be as closely matched to one of the ‘high media’ frequency diseases as possible on the following characteristics: disease fatality, symptoms and vector or mode of transmission, as described in the Centre for Disease Control database. An example of disease pair presentation can be found in Table 2.

**Table 2:** Sample Infectious Disease and Alternate Media Pair*.

Disease A	Disease B
Transmitted by a bite from an infected tick	Transmitted by a bite from an infected tick
Symptoms include malaise, anorexia, fatigue, fever, nausea, vomiting, and depression.	Symptoms include fatigue, chills, fever, headaches, rash, muscle and joint aches.
How to prevent it: spray DEET, wear long clothing (pants and longs sleeves)	How to prevent it: spray DEET, wear long clothing (pants and longs sleeves)

* Note: Disease A was Human Babesiosis, and Disease B was Lyme Disease in this example.

Participants rated the high media frequency diseases to be significantly more serious (mean = 7.8, SD = 0.174) than low media frequency diseases (mean = 6.66, SD = 0.174, on a 10-point scale) [*F* (1, 200) = 73.02, *p*<0.001]. Participants also considered high media frequency diseases to have higher disease-like status (mean = 3.04, SD = 0.088) than low media frequency diseases (mean = 2.74, SD = 0.075 on a 4-point scale) [*F* (1, 200) = 18.79, *p*<0.001]. There was no significant difference for estimations of prevalence. The individual means for each disease can be seen in Table 3.

**Table 3:** Means for Each Infectious Disease: Experiment 1.

Disease pairs (high media/low media)	Severity (/10)		Disease-like status (/4)		Prevalence (/1,000)	
	High media	Low media	High media	Low media	High media	Low media
Anthrax/Tularemia	7.77	5.79	2.31	2.63	10.5	32.7
West Nile virus/Yellow fever	8.44	7.60	3.20	2.87	44.5	40.2
Avian Flu/Hantavirus	7.81	7.18	2.89	2.77	36.5	7.5
SARS/Lassa fever	8.80	7.02	3.44	2.85	22.7	23.5
Lyme Disease/Human Babesiosis	6.35	5.69	3.38	2.59	16.3	24.9
Overall	7.81	6.66	3.04	2.74	25.2	25.7

**Figure 1:**
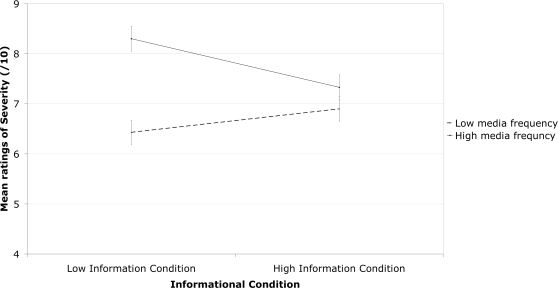
The interaction between Informational Conditions (high of low information) and Media Frequency (high or low) in estimations of severity made by undergraduate psychology participants. Error bars indicate Standard Error.

Participants assigned significantly higher ratings of seriousness to the high media frequency conditions in both the low information condition [*F*(1, 92) = 100.1, *p*<.001], and the high information condition [*F*(1, 84) = 5.79 *p*<.05]. However, the impact of media frequency was reduced in the high information condition, as evidenced by a significant interaction between information condition and media frequency [*F* (1, 200) = 67.49, *p*<0.001] in participant ratings of seriousness. This interaction between high and low media frequency diseases and informational condition can be seen in Figure 1. No such interaction was seen for estimates of disease-like status or prevalence.

Ratings of disease seriousness were strongly correlated with the frequency of print media exposure (*r* = .701, *p*<0.05). Estimates of disease-like status were moderately correlated with the frequency of print media exposure (*r* = .469, *p* = 0.17), but estimates of prevalence (*r* = .206, *p* = 0.6) were not significantly correlated with media frequency.

Participants considered the high media frequency diseases to be more serious and have higher disease-like status than the low media frequency diseases. This overall difference in perceived severity between high and low media frequency diseases was confirmed by a correlation between ratings of severity and the amount of print media exposure. The differential ratings of high and low media disorders were reduced when participants had objective information available, but only for ratings of seriousness. When provided immediate access to information pertaining to the disorder in question (in the high information condition), participants perceived the low and high frequency conditions to be closer in severity than when relying on the disease label alone.

These results to some degree support previous findings in the literature that demonstrate differential estimates for events or conditions found frequently in the media [1], [7], [9], [10], although unlike previous studies [3]–[5], we did not see a media effect on estimates of prevalence. By asking participants to rate seriousness and disease-like status, this study adds a new dimension to the current literature. These results suggest that high levels of media reporting can alter our understanding of a disease at a more holistic level than suggested by previous literature. However, it might be possible that a more medically oriented population might be less likely to show an impact of increased media exposure.

### Experiment 2

The results of Study 1 could be due to the fact that undergraduate psychology students might not be well-informed regarding infectious diseases, which could influence their judgments of severity and disease-like status. Study 2, therefore studies these same effects in a similar (in demographical terms), but more medically knowledgeable population, namely, medical students. The medical students, who presumably have more exposure to the discussion of rare diseases, more exposure to the epidemiology of diseases, and a clearer understanding of disease risk than the typical psychology undergraduate student, might be less susceptible to the impact of high levels of popular media. It is also possible that a lay population might focus on vivid and disturbing information [33] whereas a medically oriented population would focus on more objective risk factors, as seen with an expert population [5].

Participants rated the high media frequency diseases as significantly more serious (mean = 7.98, SD = 0.174), than low media frequency conditions (mean = 6.68, SD = 0.316, on a 10-point scale) [*F* (1, 164) = 12.14, *p*<0.001]. Medical students also considered high media frequency diseases to have higher disease-like status (mean = 3.14, SD = 0.112) than low media frequency diseases, (mean = 2.95, SD = 0.086, on a 4-point scale) [*F* (1, 164) = 6.502, *p*<0.05]. There was no significant difference for estimates of prevalence. The means for each disease can be seen in Table 4. It appears that even with individuals who are medically oriented, the impact of high levels of media frequency remain.

**Table 4:** Means for Each Infectious Disease: Experiment 2.

Disease pairs (high media/low media)	Severity (/10)		Disease-like status (/4)		Prevalence (/1,000)	
	High media	Low media	High media	Low media	High media	Low media
Anthrax/Tularemia	8.46	6.12	2.82	2.80	0.5	2.1
West Nile virus/Yellow fever	7.52	7.18	3.10	3.15	2.2	6.1
Avian Flu/Hantavirus	8.01	7.45	2.99	3.21	3.5	2.0
SARS/Lassa fever	8.95	6.68	3.33	3.0	2.5	6.4
Lyme Disease/Human Babesiosis	6.74	5.07	3.4	2.65	2.2	1.6
Overall	7.94	6.5	3.12	2.96	2.18	3.64

**Figure 2:**
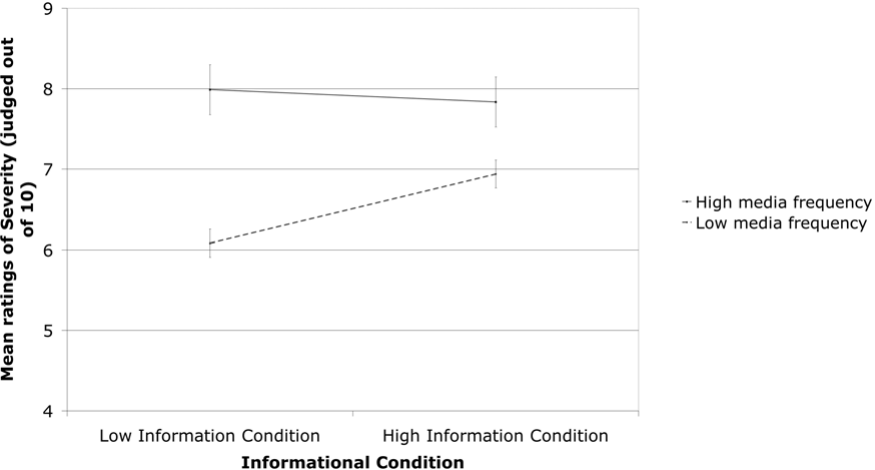
The relationship between Informational Conditions (high of low information) and Media Frequency (high or low) in estimations of severity made by medical students. Error bars indicate Standard Error.

With respect to the moderating influence of additional information, there were no significant interactions with media conditions for ratings of seriousness, disease-like status or prevalence. Figure 2 depicts the pattern of responses across informational conditions for ratings of seriousness reported by medical students. In contrast to Study 1, participants in the high information condition did not differ significantly in their ratings of seriousness, disease-like status or prevalence when compared to participants in the low information condition. That is, the concurrent presentation of objective information did not mitigate the media effect in this group. We suggest that the absence of this effect is likely due to the medical students already possessing more objective knowledge of the diseases, so the additional information did not modulate their responses as much as the undergraduate students in Study 1.

As in the first study, ratings of disease seriousness were strongly correlated with the frequency of print media exposure (*r* = .652, *p*<0.05). Frequency of print media exposure and estimates of disease-like status were moderately correlated (*r* = .44, *p* = 0.19), but were not significantly correlated for estimates of prevalence (*r* = −.12, *p* = 0.7).

There was no significant difference between ratings of seriousness and disease-like status between the two study populations. There was a significant difference between the undergraduate and medical students on estimates of prevalence [*F* (1, 88) = 7.414, *p*<0.01], with the psychology undergraduate students assigning significantly higher estimates than the medical students (psychology undergraduates = 25.0, SD = 12.2; medical undergraduates = 2.9, SD = 1.9), indicating perhaps that the medical students were better informed about epidemiology.

This study supports the trend of differential treatment of high and low media events [1], [7], [9], [10], and demonstrates that the effects found in Study 1 are not unique to a psychology undergraduate population. However, it could be possible that diseases that receive high levels of media attention are, indeed, more severe and have higher disease-like status.

### Experiment 3

Two factors that influence judgments of the severity of a particular condition: (i) the objective severity of a condition and (ii) personal knowledge of the disease based on an individual's experience in the world. Thus, an examination of the impact of media on judged severity must ensure that when one disease is judged more severe than another it is not, in fact, more severe. This final study attempts to investigate the ‘objective’ severity of a set of diseases by asking participants to judge the relative severity of a pair of disorders based on label-free disease descriptions. By eliminating prior familiarity with the disease label, we were able to distinguish the ‘objective’ severity of the disease from the participants' associated knowledge, perhaps drawn from such sources as the popular news media.

With the disease labels removed, the low media frequency diseases were seen as ‘worse’ than the high media frequency diseases (the low media frequency condition was chosen above the high media frequency condition 78% of the time [*χ* ^2^ (*df* = 9) = 30.4, *p*<0.001]. Data from Study 3 indicate that the high media frequency diseases are not objectively worse.

The results confirm that for these pairs of diseases, lacking the label or associated knowledge of the disease, the disorders that are more likely to be covered by the media are not considered to be objectively worse. Therefore, the high media frequency diseases are considered to be ‘worse’ in studies where the disease name is mentioned (as in Study 1 and 2) - it is not because the high media frequency diseases are indeed more severe.

## Discussion

This research demonstrates that individuals consider infectious diseases that receive repetitive media exposure to be more severe and have higher disease-like status than diseases of comparable objective severity that receive less media attention. Undergraduate participants will modulate their responses when information is immediately available, but medical students do not show a similar adjustment. However, while both undergraduate psychology and medical student populations rated high media frequency disorders as more serious and more disease-like, no differences in either population were found for estimates of prevalence. Since both populations rate frequent media disorders as being more severe, the effects found in this paper are not due to differential understanding of infectious disease. The perception of increased disease status and severity demonstrated in these studies represent systematic shifts based on a differential presence within popular media. In addition, ratings of severity in both populations were significantly correlated with actual print media frequency. However, data from the third experiment in this series indicate that the diseases frequently covered by the media are not, in actuality, considered to be ‘worse’ diseases than those not covered in the media.

We should, however, point out that populations used within these studies are restricted in age to groups of young adults. A study conducted by Frewer, Miles and Marsh [27] found that ratings of risk were higher in older adults, and were also higher for women than for men. Extending the study to more diverse populations, and to look at individual differences in risk perception, are possible future directions for research. However, we posit that even if mean values of risk ratings differ across populations, the general relationship between degree of media exposure and assessment should still stand.

The results presented here, and those of previous studies, speak to the media's ability not only to increase the salience of an issue [1], but to modulate an individuals' understanding of the severity of an infectious disease. The media play a critical role in shaping public opinion regarding issues, including infectious disease. Historically, most of the literature has focused on the impact of single events in terms of human effect and media coverage (i.e. the terrorist attacks on September 11^th^, e.g. [21]), and more recent, chronic threats (e.g. global warming, [1]), rather than possible disease outbreaks (SARS, avian flu). Traditional investigations into the dissociation between actual and perceived risk (e.g. [3]–[5]) have focused on estimates of prevalence, and their correlation, or lack thereof, to actual mortality statistics. With the current investigation, this form of analysis was not possible, as prospective estimations of prevalence made by participants (i.e., how many individuals will be infected with West Nile virus in the next year?) cannot be correlated with actual frequency of infection. However, through the inclusion of estimates of both severity and disease status, we have investigated the impact of media coverage on the understanding of a disease on a more holistic level.

The media function as a critical interface between the scientific community, government, and the public [9], [34] with a responsibility to strike a careful balance between raising awareness of issues of public concern and irrationally alarming the public at large [9]. Media coverage tends to be driven by issues that are rare, novel and dramatic rather than those of higher relative risk [35]. Viewers remember less than a quarter of the information and story topics [36]–[39] in a typical newscast, and news media have shifted to a more personalized presentation [40] that presents a risk as a direct threat to the viewer rather than generalized risk to a population. Since alarming content is more common in newscasts than reassuring or neutral content [41], and an estimated 11% of news articles include exaggerated claims [13], the possible impacts of disease being frequently presented in the media deserves attention.

The news media have an obligation to inform and protect the public, and have played a pivotal role in many public safety issues. However, a single incident may arouse great public concern if it is interpreted to mean that the potential risk is poorly understood [42] or difficult to control [2], as with the possibility of pandemic [43] (as in the case of Avian flu) and bioterrorism (as in the case of anthrax infection). Amplification of perceived risk can be triggered by a novel adverse event of any kind that has potential consequences for a wide range of people [5], and events that will either attenuate or amplify public concern are not easily predicted [6]. Also, if equal coverage of both frightening and reassuring information are presented in the media at a similar time, individuals will take longer to trust the reassuring information [44], and thus the introduction of high levels of media coverage of possible adverse events should be carefully considered. The data presented in this paper indicate that the concurrently presented information regarding the disease (e.g. a description of symptoms, mortality, infection rates) does decrease the difference in ratings of severity between the high and low media frequency disorders, which speaks to the need for objective and complete media reporting.

The threat of a pandemic or bioterrorism is by definition an uncertain event, one that has high personal impact [40], is highly prevalent in the media, emotionally arousing [45], personally difficult to control [3], and the reporting of which could potentially contain biased content [44]. The studies contained in this paper demonstrate that individuals will consider high media frequency disorders to be more serious and pose more of a threat than equally serious underrepresented infectious diseases. The results of these studies should add to the growing literature addressing the ability of the media to alter judgments of severity and risk. Given the results presented in this paper, it is imperative that we fully understand the effects of the media on public perceptions of disasters and disease epidemics. In this age of television and internet media it is important to consider the impact of media reporting on public perception of risk, and public health in general (e.g. [19]–[20]).
